# Chairing the internal medicine department- analysis of current state and future trends in Israel

**DOI:** 10.1186/s13584-018-0267-3

**Published:** 2018-12-19

**Authors:** Dana Zelnik Yovel, Orna Tal, Dror Dicker, Avishay Elis, Micha Rapoport

**Affiliations:** 1 Department of internal medicine “C” at Assaf Harofeh Medical Center, Zerifin, Israel; 2 Department “D” of internal Medicine at Hasharon Hospital Rabin Medical Center, Petah Tikva, Israel; 3 Department “C” of internal at Belinson Hospital Rabin Medical Center, Petah Tikva, Israel; 4 Sackler School of Medicine Tel-Aviv University, Tel Aviv, Israel

**Keywords:** Internal medicine, Chairman, Successor, Israel

## Abstract

**Background:**

Professional skills and academic records of the highest degree are essential requirements for the chairmanship of internal medicine departments. Whether the new generation and future successors of Israeli chairmen is endowed with these attributes is not known.

**Purpose:**

To determine whether there is a lack of future suitable successors for the current heads of internal medicine departments in Israel and to compare the demographic, academic and professional characteristics of the older and newer generations of department heads.

**Methods:**

An online anonymous questionnaire was nationally distributed during 2016 to all active heads of internal medicine departments in Israel (*n* = 101). First round was followed by two runs of personal phone calls to promote participation.

**Results:**

Sixty-seven (67%) of chairmen responded. The vast majority of current chairs of internal medicine departments are males (*N* = 59, 88%) over 50 years of age (*N* = 58, 86%) with established academic background with lecturer degree or higher (*N* = 57, 85%).

Only 19 (28%) of current heads assigned a future successor. Comparison of chairmen who did and did not assigned successors demonstrated that assignment of successors was associated with higher academic status (*P* < 0.02) and longer chairmanship (*p* < 0.01) but not with mean age of current chairmen (*p* < 0.08). Nevertheless, most assignments (55%) were done by chairmen in the 61 to 67 years age group. As compared to current chairmen, the designated successors have lesser academic status (*p* < 0.01) and are characterized by a higher female prevalence (*P* < 0.03).

**Conclusions:**

Significant demographic, professional and academic differences exist between the current chairs of internal medicine departments in Israeli hospitals and their future successors. This underscores the need for reassessment of the availability and requirements of this crucial position.

## Introduction

Internal medicine departments located in university affiliated medical centers are the training base for internal medicine specialists and the obligatory first phase preparation for the ensuing fellowships in the various sub-fields of internal medicine. They also serve as academic, research and teaching centers and as a professional reference point to the medical community surrounding them.

Thus, professional skills, academic records of the highest degree and administrative skills are essential requirements that heads of these departments must have in order to fulfill their multiple deeds.

The prevailing albeit undocumented impression during the past decade in Israel is that candidates that have the desired combination of academic and professional skills have become “a rare breed”, making it increasingly difficult to replace current heads of internal medicine departments with appropriate candidates. The reason (s) for the seemingly decreased candidate availability for these positions are not clear. It is possible that the ever-increasing administrative burden together with the recently growing shift of many medical practices from hospital to community significantly reduces the economic and professional rewards of hospital based position in general and head of internal medicine wards in particular. This notion is supported by Kastor et al. who conducted interviews with 44 current and former heads of internal medicine wards. They concluded that this position had lost much of its glory, in part because of increasing administrative and financial obligations that require more of the time and effort of department heads than before [1].

In order to verify this commonly held impression we determined for the 1st time whether there is a lack of future suitable candidates for the current heads of internal medicine departments in Israel. We also analyzed the characteristics of the present generation of department heads and compared them to the newer generation’s characteristics. Our novel data demonstrates that although many wards assigned in advance a future chairman, the prototype of newer heads of internal medicine department is expected to change significantly.

## Methods

### Study design

Contact information was provided by the Israeli society of internal medicine. An online anonymous questionnaire was distributed on a national level to all active heads of internal medicine departments (*n* = 101). First round was followed by two runs of personal phone calls to promote participation. Data was collected using Google DOC application. Informed consent was obtained from all participants.

### Statistical analysis

A chi test and t test was performed to compare groups. *P* value < 0.05 was considered significant.

## Results

**Fig. 1:**
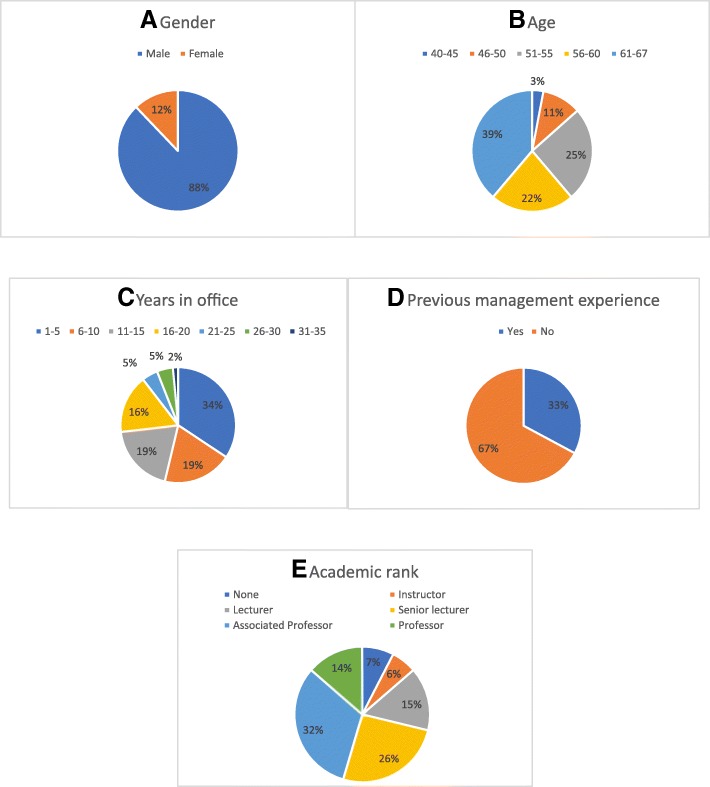
Characteristic of current chairmen. Demographic and professional characteristic of current chairmen *n* = 77 are presented: **a** Age, **b** Gender, **c** Years in office, **d** previous management experience **e** Academic Rank

Sixty seven of 101 (67%) heads of internal medicine department answered the questionnaire. Analysis of current chairmen data demonstrated that chairmen were predominantly males (88%), above the age of 50 (86%), with a significant percentage (39%) above 61 years, and the majority (85%) having an academic rank of lecturer or above. The demographic and professional characteristic of these participants appear in Fig. 1.

**Fig. 2:**
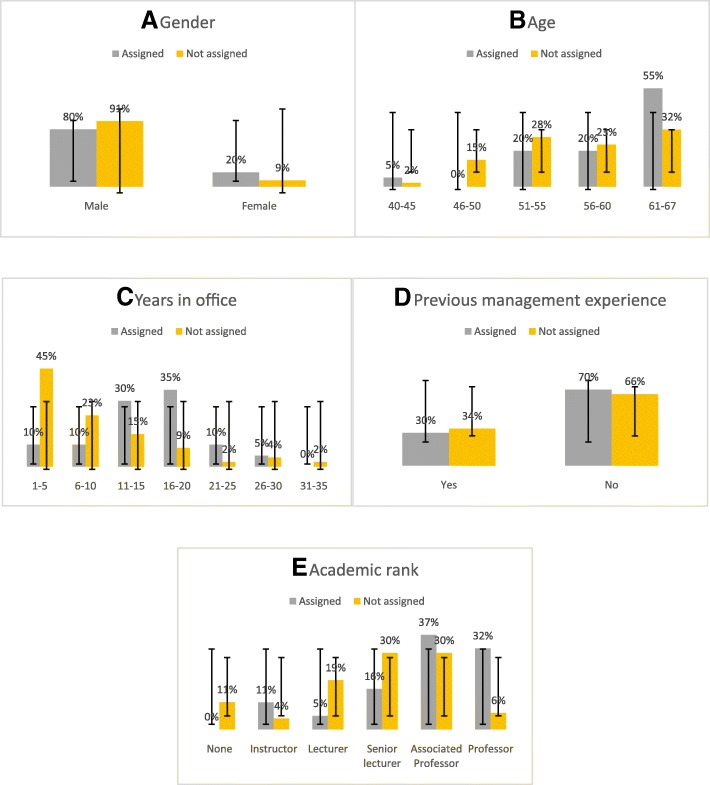
Characteristic of chairmen who assigned successors and those who did not. Demographic and professional characteristic of current chairmen who did (*n* = 19) and did not assign (*n* = 47) are presented: Data represent mean+/− Std **a** Age, **b** Gender, **c** Years in office, **d** previous management experience **e** Academic Rank.* *P* < 0.001 **P < 0.02

Nineteen (28%) current heads of departments confirmed the presence of a future successor, the majority of which 17 (89%) came from the same departments or medical centers. As demonstrated in figure2, comparison of chairmen who did and did not assigned successors demonstrated that assignment of successors was associated with higher academic status (*P* = 0.016) and longer chairmanship (*p* = < 0.01). Although the percentage of chairmen above 50 years and above 61 years was higher in the group of chairmen who assigned successors, 79 and 55% and 58 and 32% respectively, these differences did not reach statistical significance (*p* = 0.08). Nevertheless, most assignments (55%) were done by chairmen in the 61 to 67 years age group (Fig. 2).

As shown in Table 1, as compared to data of current chairmen upon their own appointment, designated successors are characterized by high prevalence of women and lower academic status. (*p* = 0.015).

**Table 1:** Demographic and professional characteristic of current chairmen of internal medicine departments and their designated successors

	Current heads n/%	Designated successors n/%	P value
Age > 50 years upon appointment	(22/65) 34%	(6/16) 37%	0.8
Gender (male)	(58/67) 88%	(11/17)65%	0.03
Academic rank of Lecturer or above upon appointment	(51/64) 80%	(8/16) 50%	< 0.01
Previous management experience	(22/67) 33%	(5/17) 29%	0.7
Subspecialty	(40/58) 69%	(7/13) 54%	0.24
Training time abroad	(35/67) 53%	(6/17) 35%	0.17

In addition, fewer designated successors had a subspecialty degree, training time abroad and previous management experience although these differences did not reach statistical significance.

In an attempt to define the ideal future successor, we asked the 47 (70%) current chairmen that did not have one nominated to indicate the most desired attributes of future successors. Forty-one (87%) of current chairmen indicated that an academic position was of a significant importance in choosing a future successor, 29 (61%) declared that it’s important for them that the candidate will come from their own hospital/department and 23 (50%) declared that experience in working abroad and previous management record are important.

We next compared the newer generation namely the assigned successor group (*N* = 19, 28%) together with the less than 5 years in office current chairmen (*N* = 27, 35%) to the older generation of department heads with more than 5 years in office.

As shown in Table 2, there were no significant differences except a sub-specialty training was significantly more prevalent in the new as opposed to the old generation of chairmen (*P* = 0.001).

**Table 2:** Demographic and professional characteristic of *older and **newer generations of department’s chairmen

	Old generation n/%	Future generation n/%	P value
Age upon appointment	(18/51) 35%	(10/30) 33%	0.85
Gender (male)	(39/44) 88%	(31/40) 77%	0.17
Academic rank of Lecturer or above upon appointment	(42/51) 82%	(19/29) 65%	0.08
Previous management experience	(12/44) 27%	(15/40) 37%	0.27
Subspecialty	(9/35) 25%	(19/34) 55%	< 0.01
Training time abroad	(25/43) 58%	(16/40) 40%	0.09

*> 5 years in office

**< 5 years in office and appointed successors

## Discussion

This work describes for the first time in Israel the professional, demographic and academic attributes of current chairmen of internal medicine wards. More importantly, it provides novel national data regarding the availability and characteristics of future candidates who will likely replace current chairmen. These data are of outmost importance as chairmanship of these wards is long lasting, the newly assigned chairmen are expected to perform as the leaders of these wards as well as the professional and academic community in the next decades. The main outcome of our work is that times are indeed changing, and the newer generation of chairmen is significantly different as compared with the current one.

Our data underscore two major differences between the current generation of chairmen and the designated successors who have lesser academic degree and as a group have a higher prevalence of females.

Together with the observation that current heads with less than 5 years in office have as group lower academic ranks, they do support the currently held belief that these positions have been fulfilled with candidates of a lesser academic achievements, and this trend is likely to continue. It should be noted however, that the differences in academic rank became non-significant when not only successors but rather the new generation of chairmen, composed of successors and chairmen with less than 5 years in office were compared to the older generation chairmen (> 5 years in office). Nevertheless, these 2 groups still differed in their professional attributes namely sub-specialty training which was significantly more prevalent in the new generation of chairmen.

The importance of higher academic rank as an essential requirement for the chairmanship position is further underscored by the clearly expressed opinion of the vast majority current chairmen who established academic achievement as a first priority in choosing their future successor.

The reasons underlying these persistent gradual changes are not fully clear. Most probably, the increased administrative burden combined with the ever widening field of ambulatory medicine reduced the attractiveness of hospital based positions and provided increasingly more rewarding options out of hospital both economically and professionally [2, 3] .

The higher prevalence of females among the new generation of department heads can be explained by the persistent increase of female predominance among medical students reaching over 60%, according to Tel Aviv Sackler faculty of medicine data base. As well as the current composition of medical staff in the general medicine wards [4] allowing for more female candidates to compete for chairmanship positions [5]. This change takes place in spite of their higher levels of work-family conflict resulting in higher double burden than men [6].

Taken together these evolving trends initiate and maintain the gradual change observed in the attributes of new chairmen of internal medicine wards.

These observed trends are not limited to the Israeli medical community and most likely represent a worldwide trend. Similar but not identical findings were reported by Rayborn et al. demonstrating increased prevalence of women among 1^st ^time new chairs in 29 years perspective on recruitment and retention of department chairs in at U.S. medical schools [7]. These new chairs were however older as compared to former ones. Similarly to our findings, Heitz et al, in a survey of current academic chairmen of emergency departments, found that, academic experience including scholarly productivity, peer-reviewed publication, faculty development, and graduate medical education, was the most important skill to obtain prior to becoming a chair, while hospital governance and cross-departmental collaboration skills can be obtained once in the role [8]. Finally, after interviewing 44 current and former chairs, deans, division chiefs, and hospital directors in the U.S., Kastor concluded that “the job of chair of a department of medicine, once seen as the apex in the career of an academic internist, has lost much of its allure, in part because of increasing administrative and financial obligations that require more of the time and effort of chairs than formerly” [1].

Our findings, in particularly those regarding the attributes of designated successors, should be interpreted with caution as much as it is not known what will be the age and academic rank of the designated successors upon assuming office. In addition, current chairmen can suggest and designate a potential candidate for chairmanship but by no mean have the official jurisdiction to appoint their successors or accurately predict when and if succession will effectively take place. The decision regarding succession is done by an official committee composed by several representatives who may prefer other candidates rather the designated successors. Thus, although there is no available data in that regard, it is conceivable that only a portion of the designated successors ultimately are appointed.

Nevertheless, our data together with similar trends observed worldwide provide valuable insights regarding the changing world of internal medicine chairmanship. Most importantly, it strongly suggests that it is mandatory that the leaders of public health who are in charge of planning future health policies and medical education including ministry of health, deans of medical schools, the internal medicine professional associations and hospital directors should all be aware of these change and act accordingly. Indeed, due to the current shortage of senior academic positions in the affiliated teaching wards the Tel Aviv faculty of medicine has recently initiated an academic hospital affiliation allowing non-fully academic wards to participate in medical students teaching.

## Conclusion

Significant demographic, professional and academic differences exist between the current chairs of internal medicine departments in Israeli hospitals and their future successors. These significant differences underscore the need for reassessment of the needs and requirements of this crucial position.
